# Treatment with the Hyaluronic Acid Synthesis Inhibitor 4-Methylumbelliferone Suppresses SEB-Induced Lung Inflammation

**DOI:** 10.3390/toxins5101814

**Published:** 2013-10-17

**Authors:** Robert J. McKallip, Harriet F. Hagele, Olga N. Uchakina

**Affiliations:** Division of Basic Medical Sciences, Mercer University School of Medicine, 1550 College St, Macon, GA 31207, USA; E-Mails: harriet.flora.hagele@live.mercer.edu (H.F.H.); uchakina_o@mercer.edu (O.N.U.)

**Keywords:** staphylococcal enterotoxin B, acute lung injury, hyaluronic acid

## Abstract

Exposure to bacterial superantigens, such as staphylococcal enterotoxin B (SEB), can lead to the induction of acute lung injury/acute respiratory distress syndrome (ALI/ARDS). To date, there are no known effective treatments for SEB-induced inflammation. In the current study we investigated the potential use of the hyaluronic acid synthase inhibitor 4-methylumbelliferone (4-MU) on staphylococcal enterotoxin B (SEB) induced acute lung inflammation. Culturing SEB-activated immune cells with 4-MU led to reduced proliferation, reduced cytokine production as well as an increase in apoptosis when compared to untreated cells. Treatment of mice with 4-MU led to protection from SEB-induced lung injury. Specifically, 4-MU treatment led to a reduction in SEB-induced HA levels, reduction in lung permeability, and reduced pro-inflammatory cytokine production. Taken together, these results suggest that use of 4-MU to target hyaluronic acid production may be an effective treatment for the inflammatory response following exposure to SEB.

## 1. Introduction

Exposure to staphylococcal enterotoxin B (SEB) resulting from infection with *Staphylococcal aureus* can result in life threatening complications due to activation of up to 40% of naïve T and possibly NKT cells [1,2,3,4]. This exaggerated response leads to the production of a number of pro-inflammatory cytokines including IL-1β, IL-2, IL-6, IFN-γ, and TNF-α [5,6], which can lead to endothelial cell injury, acute lung injury (ALI), acute respiratory distress syndrome (ARDS), and vascular collapse (shock) [7]. Due to its potential to cause widespread disease, its universal availability and ease of production, and dissemination SEB is currently listed by the Centers for Disease Control and Prevention (CDC) as a category B select agent. Currently, there are no known effective treatments for these conditions [8]. 

Modulation of the extracellular matrix can play an important role in the regulation of the inflammatory response. For example, a number of reports demonstrate that increased production hyaluronic acid is associated with various inflammatory conditions [9,10,11]. Under normal non-inflammatory conditions, hyaluronic acid exists primarily in its high molecular weight form (HMW-HA). However, under inflammatory condition low molecular weight hyaluronic acid (LMW-HA) accumulates [12]. Additional evidence suggests that LWM-HA has pro-inflammatory activity while HWM-HA has anti-inflammatory properties. 

In recent work examining a possible role of HA in SEB-induced vascular damage we revealed that following SEB exposure, there was an increase in the level of HA in the lungs and that treatment with a HA blocking peptide led to a significant reduction in SEB-induced lung injury [13]. In the current study, we tested the hypothesis that inhibition of hyaluronic acid production will lead to a reduction in lung inflammation following exposure to SEB. To test this hypothesis we used the hyaluronic acid synthesis inhibitor 4-MU, and examined its effect on SEB-induced acute lung inflammation (ALI). Knowledge gained from this study will advance our understanding of the role of HA in SEB-mediated vascular damage and may ultimately lead to significantly improved treatment of symptoms associated with SEB exposure.

## 2. Results and Discussion

### 2.1. 4-MU Inhibits SEB-Induced Leukocyte Proliferation and Cytokine Production *in Vitro*

**Figure 1:**
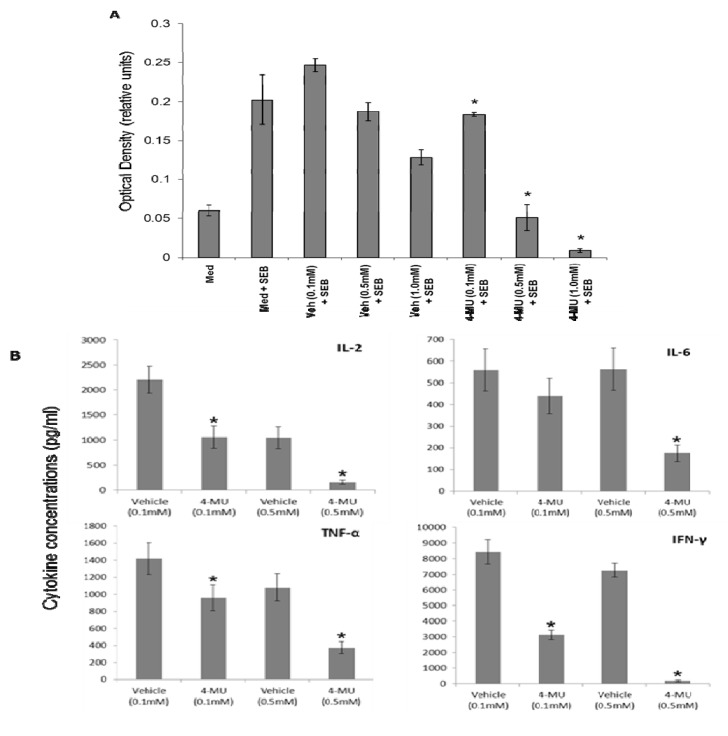
4-MU inhibits SEB-induced leukocyte proliferation and cytokine production *in vitro*. Spleen cells from C57BL/6 mice were treated with 4-MU (0.1, 0.5, and 1.0 mM) or vehicle control (DMSO) and then stimulated with SEB (2 μg/mL). The effect of 4-MU on the proliferative response and cytokine production was determined 48 h later by MTT assay (**A**) and cytometric bead array (**B**), respectively. Asterisks indicate statistically significant difference when compared with vehicle controls, *p* ≤ 0.05.

Exposure to SEB leads to activation of lymphocytes, which is characterized by elevated proliferation as well as increased production of inflammatory cytokines. Studies were conducted to determine whether culturing lymphocytes with 4-MU had an effect on SEB-induced proliferation or cytokine production. To this end, spleen cells were cultured with various concentrations of 4-MU (0.1, 0.5, or 1.0 mM) or vehicle control and stimulated with SEB. The effects of 4-MU on SEB-induced proliferation were determined 48 h later by MTT assay and revealed that treatment with 4-MU at concentrations as low as 0.1 mM significantly reduced the proliferative response (Figure 1A). Next, the effects of 4-MU on cytokine production were examined by measuring the levels of cytokines in the culture supernatants following 48 h of culture using a cytometric bead assay. The results demonstrated that treatment with 4-MU significantly inhibited SEB-induced cytokine production (Figure 1B). Taken together, these results demonstrate that culturing spleen cells with 4-MU significantly inhibits the inflammatory response to SEB.

### 2.2. 4-MU Treatment Leads to Increased Apoptosis in SEB-Exposed T lymphocytes *in Vitro*

**Figure 2:**
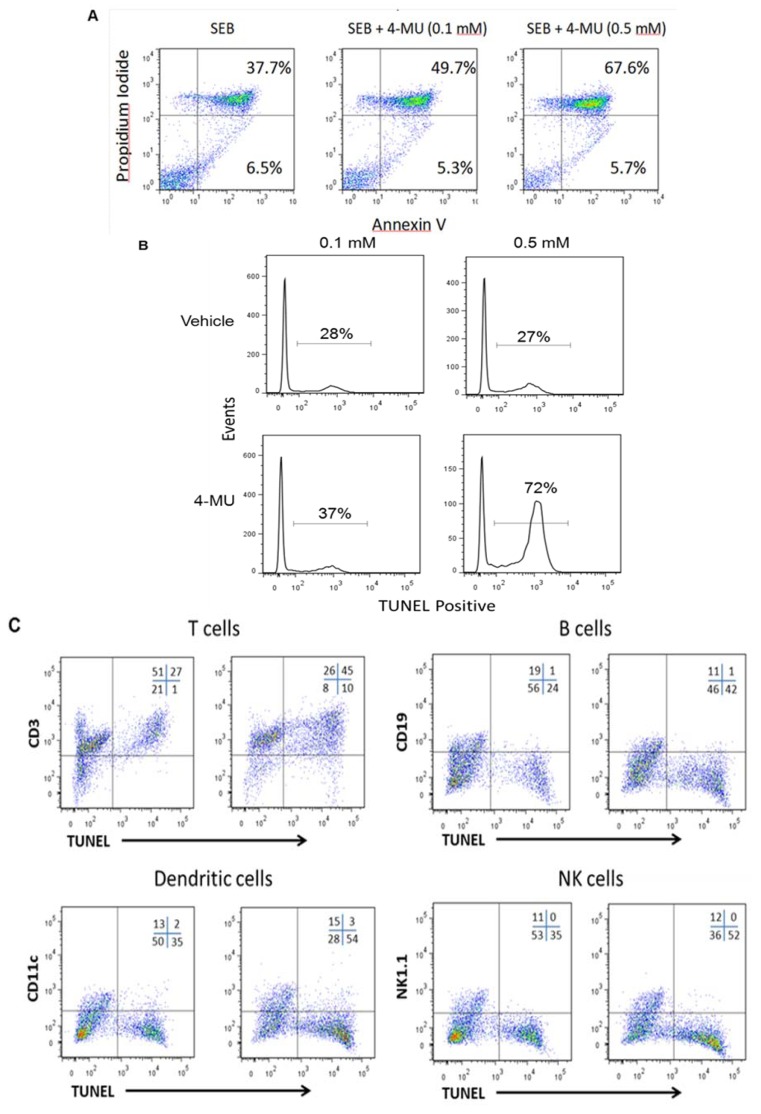
4-MU treatment leads to increased apoptosis in SEB-exposed leukocytes *in vitro*. Spleen cells from C57BL/6 mice were treated with 4-MU (0.1, and 0.5 mM) or vehicle control (DMSO) and then stimulated with SEB (2 μg/mL). The effect of 4-MU on SEB-induced apoptosis was determined 48 h later by Annexin V/PI (**A**) and TUNEL (**B**) assays, respectively. The level of apoptosis in individual immune cell subsets was determined by staining the spleen cells with phenotype-specific fluorescently-labeled mAbs followed by TUNEL staining (**C**).

Mechanistically, 4-MU treatment has been shown to induce cell death in various cancers as well as in through the induction of apoptosis through and effect on HA production [14,15,16]. Furthermore, previous results from our laboratory demonstrated that SEB exposure leads to increased production of HA in the lungs and in separate studies we demonstrated an important role of CD44 in lymphocyte activation-induced cell death (AICD), suggesting the possibility that SEB-induced HA production may protect immune cells from apoptosis through binding CD44 [2,13]. This possibility is further supported by other studies that demonstrated a protective role of CD44 in lymphocyte survival [17,18]. Therefore, we examined whether treatment of SEB-exposed spleen cells with 4-MU had an effect on the induction of apoptosis. To this end, spleen cells were exposed to SEB and then treated with 4-MU (0.1 and 0.5 mM) or vehicle control. The levels of apoptosis were determined 48 h later by Annexin V/PI (Figure 2A) and TUNEL (Figure 2B) assays. The results demonstrated that treatment with 4-MU at concentrations as low as 0.1 mM led to an increase in the levels of apoptosis suggesting that 4-MU may act to suppress the immune response to SEB through the induction of apoptosis. This effect was specific for SEB-exposed spleen cells as no significant increase in apoptosis was seen in niave spleen cells following 4-MU treatment (data not shown). In addition, we examined whether treatment with 4-MU had an effect on apoptosis in specific leukocytes subsets. Spleen cells were exposed to SEB and then treated with 4-MU (0.5 mM) or vehicle control. The effect on the specific leukocyte subsets was determined 48 h later by staining the cells with fluorescently-labeled phenotype-specific mAbs followed by TUNEL staining. The results demonstrated that 4-MU treatment had little effect on B cell, dendritic cells, or NK cells, but specifically induced apoptosis in the T cell populations (Figure 2C). 

### 2.3. 4-MU Treatment Suppresses SEB-Induced Hyaluronic Acid Synthase Expression and Accumulation of Soluble HA in the Lungs

**Figure 3:**
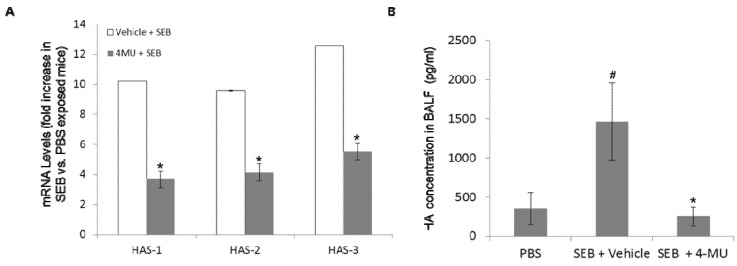
4-MU treatment suppresses SEB-induced hyaluronic acid synthase expression and accumulation of soluble HA in the lungs. The effect of 4-MU on soluble HA levels in the lungs of SEB-exposed mice was determined by treating the mice with 4-MU (450 mg/mouse i.p.) or vehicle control (5% gum arabic) one day prior and on the day of SEB exposure (20 µg/50 μL PBS). The levels of lung hyaluronic acid and mRNA levels of HAS were determined 24 h later. HAS mRNA levels in whole lung extracts were determined by real-time RT-PCR (**A**). Hyaluronic acid levels in bronchoalveolar lavage fluid (BALF) were determined by ELISA (**B**). Asterisks indicate statistically significant difference when compared with the levels from vehicle-exposed mice, *p* ≤ 0.05. Number sign indicates statistically significant difference when compared to PBS exposed mice, *p* ≤ 0.05.

Previously, we reported that exposure to SEB led to an increase in the levels of soluble HA in the lungs of mice and that treatment with a HA blocking peptide could protect mice from SEB-induced lung injury [13]. It has been reported that 4-MU can reduce hyaluronic acid production through inhibition of the mRNA expression of hyaluronic acid synthases or through the depletion of UDP-GlcUA which are essential for HA synthesis [13,19,20,21]. In the current study we examined whether treatment with 4-MU had any effect on the hyaluronic acid synthase expression (HAS) or soluble HA levels in the lungs of SEB-exposed mice. Initially, experiments were conducted to examine the effects of 4-MU treatment on the expression of HAS in the lungs of SEB-exposed mice. To date three isoforms of hyaluronic acid synthase (HAS-1, HAS-2, and HAS-3) have been identified [22]. Exposure to SEB led to increased expression of all three isoforms, which was significantly inhibited by 4-MU treatment (Figure 3A). Next, we examined the effects of 4-MU on soluble levels of HA in the lungs of SEB-exposed mice. The results demonstrated that exposure to SEB led to significantly elevated levels of soluble HA in the lungs and that this increase was significantly reduced by treatment with 4-MU (Figure 3B). Taken together, these results demonstrated that SEB exposure alters lung levels of soluble HA possibly through increased expression of HAS mRNA and that treatment with 4-MU can significantly inhibit the accumulation of HA by reducing HAS mRNA expression. 

### 2.4. 4-MU Treatment Protects Mice from SEB-Induced Acute Lung Injury

**Figure 4:**
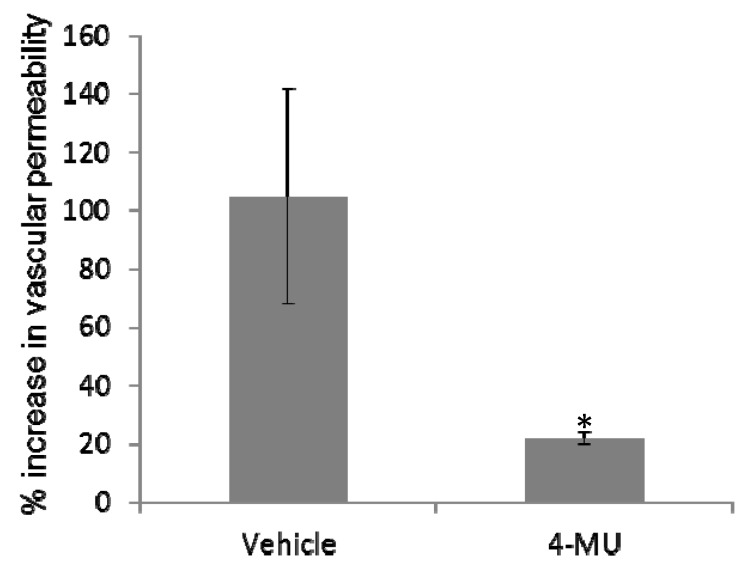
The effect of 4-MU on SEB-induced vascular permeability *in vivo* was determined by exposing mice to SEB, as described in the Material and Methods section, and treating the mice with 4-MU (450 mg/mouse i.p.) or Vehicle (5% gum arabic i.p.) one day prior, and on the day of, SEB exposure. Vascular permeability was determined as described Materials and Methods. Asterisks indicate statistically significant difference when compared with the Vehicle-treated controls, *p* ≤ 0.05.

SEB exposure can lead to acute lung injury, which is characterized by an increase in vascular permeability [16,23]. After demonstrating that treatment with 4-MU was effective at reducing the levels of HA in the lungs of SEB-exposed mice, we examined the effectiveness of targeting soluble hyaluronic acid synthesis using 4-MU as a mean to prevent SEB-induced increase in vascular permeability *in vivo*. Mice were treated one day prior to, and on the day of, SEB exposure with 4-MU (450 mg/kg) or vehicle control and then exposed to PBS or SEB (20 µg/50 μL PBS). The effect of 4-MU on the SEB-induced inflammatory response was examined by determining levels of vascular permeability in the lungs (Figure 4). The results demonstrate that vascular permeability is significantly greater in SEB-exposed mice treated with vehicle control than in corresponding PBS-exposed mice. The degree of vascular permeability in the SEB-exposed treated with 4-MU was significantly less than that seen in SEB-exposed mice treated with vehicle. Specifically, the OD reading increased from 0.082 ± 0.026 in the PBS-exposed vehicle treated mice to 0.168 ± 0.030 in the SEB-exposed vehicle treated mice which represented a 105% increase in vascular permeability following SEB exposure. In contrast the OD reading in the 4-MU treated mice increased from 0.079 ± 0.007 in the PBS-exposed mice to 0.100 ± 0.002 in the SEB-exposed mice representing a 24% increase in vascular permeability compared to the PBS exposed mice. Together, these results suggest that inhibition of hyaluronic acid synthesis by treating mice with 4-MU can lead to significant protection from SEB-induced lung injury. Furthermore, these results demonstrate that 4-MU treatment alone did not lead to a significant toxicities in the lungs as the vascular permeability in the PBS-exposed mice was not significantly increased following treatment with 4-MU when compared to vehicle-treated mice.

### 2.5. 4-MU Treatment Suppresses SEB-Induced Inflammatory Cytokine Production in the Lungs

**Figure 5:**
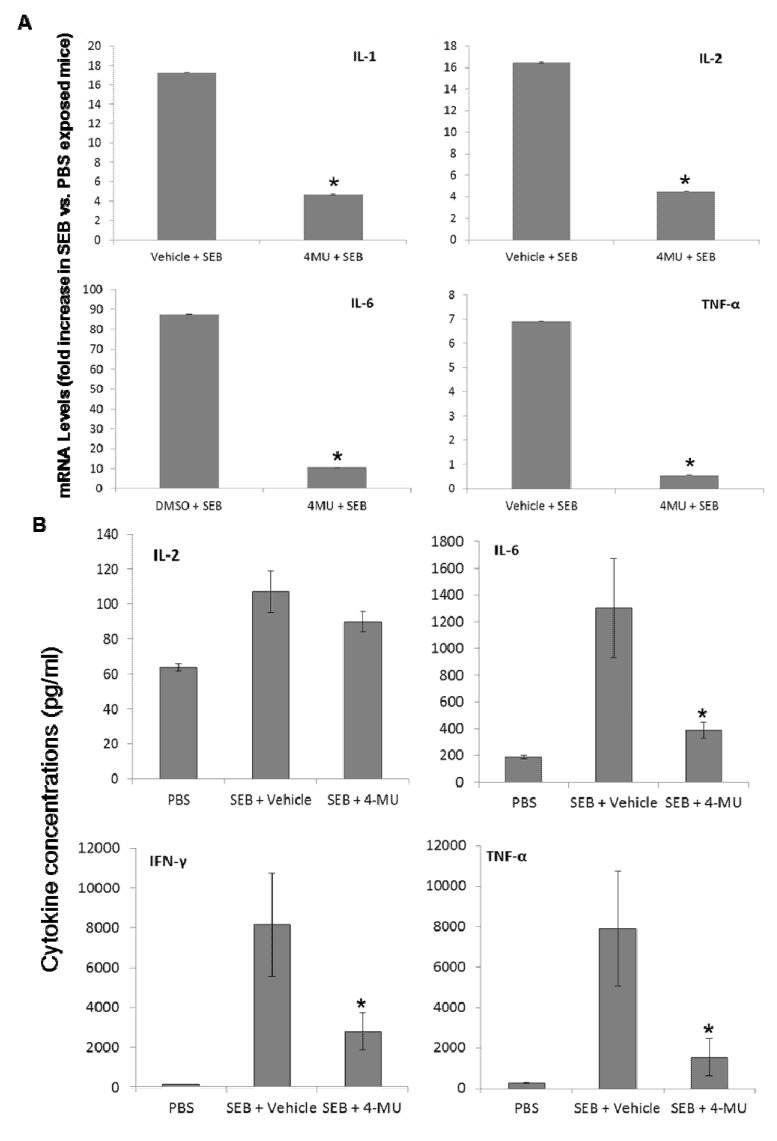
4-MU treatment suppresses SEB-induced inflammatory cytokine production in the lungs. The effect of 4-MU on SEB-induced inflammatory cytokine production *in vivo* was determined by treating the mice with 4-MU (450 mg/mouse i.p.) or vehicle (300 µL 5% gum arabic/mouse i.p.) one day prior to, and on the day of, SEB exposure. Following 4-MU treatment mice were exposed to SEB or PBS, as described in the Material and Methods section. The levels of BALF cytokine protein levels and total lung cytokine mRNA and were determined 24 h later. Cytokine mRNA levels in whole lung extracts were determined by real-time RT-PCR (**A**). The protein levels of cytokines in BALF were determined using a cytokine bead array (**B**). Asterisks indicate statistically significant difference when compared to the cytokine levels from SEB-exposed vehicle-treated mice, *p* < 0.05.

A hallmark feature of SEB-induced acute lung injury is the activation of immune cells leading to a cytokine storm characterized by the release of large quantities of pro-inflammatory cytokines, such as IL-1β, IL-2, IL-6, IFN-γ, and TNF-α [5,16,23]. Therefore, the ability to prevent or treat the pathologies associated with SEB exposure relies on controlling the levels of SEB-induced cytokines. Experiments were set up to explore the potential use of 4-MU to reduce the SEB-induced increase in cytokine levels in the lung. To this end, groups of mice were treated with either vehicle control or 4-MU (450 mg/mouse i.p.) one day prior, and on the day of, SEB exposure. Following 4-MU treatment the mice were exposed to either PBS or SEB (20 µg/50 µL PBS i.n.). BALF and whole lung tissue were harvested 24 h later. Cytokine mRNA levels in whole lung extract were determined by real-time RT-PCR (Figure 5A). Cytokine protein levels in the BALF were determined by cytometric bead array (CBA) (Figure 5B). The results demonstrate that exposure of vehicle-treated mice to SEB led to an increase in the expression of a number of cytokines, including IL-1β, IL-2, IL-6, IFN-γ, and TNF-α, which are all reported to play a role in ALI/ARDS [13,23]. In comparison, treatment of mice with 4-MU led to a significant reduction in the SEB-induced increase in all cytokines measured. 

## 3. Experimental Section

### 3.1. *In Vitro* Proliferation Assay

The spleens were harvested from euthanized C57BL/6 mice and placed into 10 ml of RPMI 1640 (Gibco Laboratories, Grand Island, NY, USA) supplemented with 5% FCS, 10 mM HEPES, 1 mM glutamine, 40 μg/mL gentamicin sulfate, and 50 μM 2-mercaptoethanol, referred to as complete medium. The spleens were prepared into a single cell suspension using a laboratory homogenizer, washed twice, and adjusted to 5 × 10^6^ /mL in complete medium. The splenocytes (5 × 10^5^ in 100 μL/well) were cultured in 96 well flat-bottomed plates in the presence of various concentrations of 4-MU and either left unstimulated or stimulated with 2 µg/mL SEB for 48 h. The relative number of viable cells was determined using the MTT assay following the manufacturer’s (Trevigen, Gaithersburg, MD, USA) instructions. Briefly, 10 µL of MTT reagent was added to each well and the plates were incubated at 37 °C for 4 h. Next, 100 μL of detergent was added to each well and the plates were incubated in the dark for 4 h, after which the absorbance at 570 nm was determined using a microplate reader. 

### 3.2. Bead Array Analysis of Cytokine Levels

Cytokine assessment was carried out using the BD Cytometric Bead Array (CBA) (BD Bioscience, San Jose, CA, USA) for simultaneous detection of multiple cytokines (IL-2, IL-6, IFN-γ, and TNF-α) in cell supernatants and bronchoalveolar lavage fluid (BALF) of PBS or SEB exposed mice. Briefly, test samples and PE detection antibody were incubated with capture beads for 2 h, in the dark, at room temperature. After which, all unbound antibodies were washed and the beads were resuspended in 300 μL PBS. The cytokine levels were analyzed using a BD FACSAriaII cell sorter. Cytokine levels were calculated using the FCAP Array Software v3.0 (BD Bioscience, San Jose, CA, USA).

### 3.3. Quantification of Apoptosis

Spleen cells (5.0 × 10^6^ cells/well) were cultured in 24-well plates in the presence of various concentrations of 4-MU (0.1 or 0.5 mM) or vehicle control and then stimulated for 48 h with SEB (2 µg/mL). Next, the cells were harvested, washed twice in PBS, and analyzed for the induction of apoptosis using the TUNEL, and Annexin V/PI methods. To detect apoptosis using the TUNEL method the cells were washed twice with PBS and fixed with 4% *P*-formaldehyde for 30 min on ice. The cells were next washed with PBS, permeabilized by adding 70% EtOH for 20 minutes, and incubated with FITC-dUTP and TdT (Promega, Madison, WI, USA) for 1 h at 37 °C and 5% CO_2_. To detect apoptosis using Annexin/PI, the samples were stained with FITC labeled Annexin V and PI. The cells were incubated for 15 min at room temperature and subsequently analyzed by flow cytometric analysis. The samples were analyzed using a flow cytometer (FACSAria II, BD Biosciences, San Jose, CA, USA). In all experiments, 10,000 cells were analyzed using forward/side-scatter gating. The level of apoptosis in the individual immune cell subsets spleen cells was analyzed by flow cytometric analysis using fluorescently-labelled mAb specific for T cells (CD3), B cells (CD19), dendritic cells (CD11c), and NK (NK1.1). The level of apoptosis in activated *vs.* naïve cells was analyzed using mAbs specific for CD69. The level of apoptosis in these subsets was determined by TUNEL staining. 

### 3.4. Hyaluronic Acid Quantification

HA levels in BALF of PBS or SEB exposed mice were measured by an ELISA-like assay according to the manufacturer’s protocol (Echelon Biosciences, Salt Lake City, UT, USA).

### 3.5. RNA Isolation and Real-Time RT-PCR Analysis

Total RNA was isolated from a single cell suspension of splenocytes or from whole lung using the RNeasy Mini Kit (Qiagen, Valencia, CA, USA). RNA concentration and integrity was determined spectrophotometrically. cDNA was synthesized by reverse transcription of 50 ng total RNA using the High Capacity cDNA Reverse Transcriptase Kit (Applied Biosystems, Carlsbad, CA, USA). Real-time PCR was performed using a SYBR Green PCR kit (Applied Biosystems). Amplifications were performed and monitored using an ABI 7300 real-time PCR system (Applied Biosystems). The gene-specific primers for β-actin have been previously described (17). In addition the following primers were used: IL-1β primers 5'-GAAATGCCACCTTTTGACAGTG-3' and 5'-CTGGATGCTCTCATCAGGACA-3'; IL-2 primers 5'-TGATGGACCTACAGGAGCTCCTGAG-3' and 5'-GAGTCAAATCCAGAACATGCCGCAG-3'; TNF-α primers 5'-CCAGTGTGGGAAGCTGTCTT-3' and 5'-AAGCAAAAGAGGAGGCAACA-3'; HAS-1 primers 5'-ACCTCACCAACCGAATGCTT-3' and 5'-GAAGGAAGGAGGAGGGCG-3'; HAS-2 primers 5'-TGAGTACAAAGAGGTTCGTTCAAGTT-3' and 5'-ATTGTCAGGGTGTGTTTGTTTCC-3'; HAS-3 primers 5'- CTACTTTGTAGCTGCCCAGAATACTG-3' and 5'-GAGTAC AAAAAACAGCACCGGAAT-3'; HYAL-1 primers 5'-GGCCTACCTAGGACTTCCTCAA-3' and 5'-CTATTCCCGTGACTGTGCCTAT-3'; HYAL-2 primers 5'-GACCTCAACTACCTGCAGAAGC-3' and 5'-CCTTATAGTTCCG TTGGCACTG-3'; HYAL-3 primers 5'-GGAGCATTCACATCCTCTACCT-3' and 5'-GTCGTCCAGAGACAGGAATCTC-3'. The threshold cycle (CT) method was used for relative quantification of gene transcription in relation to expression of the internal standard β-actin. Fold changes of mRNA levels in SEB-stimulated immune cells relative to unstimulated cells was determined using the 2^−ΔΔCt^ method [24]. 

### 3.6. Quantification of Vascular Permeability

Vascular leakiness was studied by measuring the extravasation of Evan’s blue, which when given i.v. binds to plasma proteins, particularly albumin, and following extravasation can be detected in various organs as described previously [25]. Vascular leak was induced by injection of SEB, as previously described [13,23]. Briefly, groups of five mice were injected i.n. with SEB (20 µg/50 µL PBS) or PBS (50 μL). The mice were exposed to SEB for 24 h. Two hours prior to harvesting the lungs, the mice were injected i.v. with 0.1 mL of 1% Evan’s blue in PBS. After two hours, the mice were exsanguinated under anesthesia, and the heart was perfused with heparin in PBS as described previously [7]. The lungs were harvested and placed in formamide at 37 °C for 16 h. The Evan’s blue in the organs was quantified by measuring the absorbance of the supernatant at 650 nm with a spectrophotometer. In experiments examining the effect of 4-MU on SEB-induced vascular permeability, mice were treated one day prior to, and on the day of, SEB exposure with vehicle or 4-MU (450 mg/mouse, i.p.). The vascular permeability seen in SEB-exposed mice was expressed as percent increase in extravasation when compared with that of PBS-treated controls and was calculated as: (optical density of dye in the lungs of SEB-exposed mice)-(optical density of dye in the lungs of PBS-treated controls))/(optical density of dye in the lungs of PBS-treated control) × 100. Each mouse was individually analyzed for vascular permeability, and data were expressed as mean ± SEM percent increase in vascular permeability in SEB-exposed mice when compared to that seen in PBS-treated controls [13,23].

### 3.7. Statistical Analysis

Student’s *t*-test or ANOVA was used to determine statistical significance and *p* < 0.05 was considered to be statistically significant.

## 4. Conclusions

Exposure to bacterial superantigens, such as SEB, can lead to the induction of acute lung injury/acute respiratory distress syndrome (ALI/ARDS). Currently, there are no effective treatments for the resulting inflammatory response. Results from our previous study demonstrated an important role of hyaluronic acid in development of SEB-induced ARDS/ALI [13]. Specifically, we showed that SEB exposure leads to increased levels of hyaluronic acid in the lungs and that targeting hyaluronic acid using Pep-1 led to a significantly attenuated response to SEB *in vitro* and *in vivo*. In the current study, we explored an alternative approach to reduce hyaluronic acid levels in the lungs following SEB-exposure by targeting hyaluronic acid synthesis using 4-MU. The protective effects of 4-MU treatment were characterized by reduced expression of HAS-1, HAS-2, and HAS-3, and reduced levels of HA in the lungs of SEB-exposed mice. Furthermore, 4-MU treatment led to a reduction in SEB-induced lung permeability, and reduced cytokine production. Together, the findings of this study suggest that targeting hyaluronic acid synthesis might be a novel target for the treatment or reduction of SEB-induced lung injury. 
